# Reproducibility of A Non-Quantitative Food Frequency Questionnaire (62-Item FFQ-6) and PCA-Driven Dietary Pattern Identification in 13–21-Year-Old Females

**DOI:** 10.3390/nu11092183

**Published:** 2019-09-11

**Authors:** Ewa Niedzwiedzka, Lidia Wadolowska, Joanna Kowalkowska

**Affiliations:** Department of Human Nutrition, University of Warmia and Mazury in Olsztyn, Sloneczna 45F, 10-718 Olsztyn, Poland

**Keywords:** FFQ, test–retest, reproducibility, reliability, dietary assessment, dietary pattern, kappa coefficient, girls, women

## Abstract

The aim of this study was to evaluate the test–retest reproducibility of a non-quantitative food frequency questionnaire (acronym: 62-item FFQ-6) and the possibility of identifying dietary patterns (DPs) in 13–21-year-old females. The study involved 97 females within three age groups: 13–15, 16–18, and 19–21 years, including 31, 38, and 28 subjects, respectively. The questionnaire was completed twice with a two-week interval (test and retest). For the total sample, using a principal component analysis (PCA), two similar PCA-driven DPs (DP1 and DP2) were identified separately from test data and retest data, considering two sets of input variables. 60-item-DP1 and 60-item-DP2 were identified after excluding two items—vegetables and fruits in general—due to including single items of various kinds of vegetables and fruits. After an aggregation of some items of the questionnaire, 25-item-DP1 and 25-item-DP2 were identified. The kappa statistic (test vs. retest) in the total sample averaged at 0.52 (0.32–0.72 for food items), while within age groups, it averaged at 0.41, 0.53, and 0.65, respectively. The percentage of subjects classified into the same food frequency category (test vs. retest) in the total sample averaged at 68% (51%–89% for food items), while within age groups, it averaged at 60%, 68%, and 77%, respectively. The Spearman correlations between dietary pattern scores (test vs. retest) in the total sample were: 0.84 (within age groups 0.83, 0.81, and 0.78, respectively) for 60-item-DP1, 0.68 (within age groups 0.24, 0.79, and 0.76, respectively) for 60-item-DP2, 0.76 (within age groups 0.56, 0.82, and 0.89, respectively) for 25-item-DP1, and 0.48 (within age groups 0.40, 0.57, and 0.53, respectively) for 25-item-DP2 (*p* < 0.05 for all). In conclusion, the test–retest reproducibility of the 62-item FFQ-6 was good or very good for most food items, with a tendency to be higher in older age groups of females under study. Due to the acceptable-to-good reproducibility of dietary pattern identification, the use of a 62-item FFQ-6 to describe the overall diet of young Polish females can be recommended.

## 1. Introduction

An assessment of dietary intake is still a topic of interest due to the causal relation of many diseases with diet and the possibility of identifying individuals and subpopulations at risk of inadequate food consumption [1]. There is no gold-standard method for dietary intake assessment. The most commonly used are three methods: food record, 24-hour dietary recall, and food frequency method using food frequency questionnaires (FFQs) [2,3,4]. These methods are characterized by various levels of validity and reproducibility since the validity and reproducibility of any dietary method is a function of the measurement errors and uncertainty resulting from true variability in daily food consumption and modulating factors such as sex, age, personality, education level, family affluence, national wealth, tradition, religion, and seasonality in food supply [2,5]. 

FFQs are often used in dietary assessment because they are practical, easy, and quick to administer, relatively inexpensive and less engaging the respondent, and also better at describing the usual diet than other methods [1,5,6,7]. The main possible respondent-related errors, which can be attributed to FFQ use include skipping or adding food reported or inadequate assessment of the frequency and/or quantity of food consumed [2,5]. Despite this, it was shown that FFQs can be a reliable tool to assess selected food consumption or selected nutrient intake and to describe the whole diet, including dietary patterns [7,8,9,10,11]. Identifying dietary patterns (DPs) has been widely used in various subpopulations across the world to find association of DPs with health outcomes [7,12,13,14].

All newly developed or modified FFQs have to be checked for reproducibility and relevance in respect to the population under study [1,5]. In Poland, there are a few semi-quantitative food frequency questionnaires which have been developed for selected nutrients and validated in young adults, i.e., calcium [15], vitamin D [16], iron [17], iodine [18], folate [19], and zinc [20]; a short non-quantitative food frequency questionnaire (FIVeQ) developed to measure food intake variety in older people [21]; a full semi-quantitative food frequency questionnaire (165-item FFQ) compared with a 3-day food record in young females [22,23]; and a non-quantitative food frequency questionnaire (KomPAN®) whose reproducibility was assessed in adolescents and adults [24]. The 165-item FFQ is difficult to apply due to a large number of detailed questions, although it was successfully used to study dietary patterns and adverse health outcomes in adolescents [13]. The KomPAN® allows diet to be described in terms of ‘healthy’ and ‘unhealthy’ dietary behaviors with pre-defined diet quality scores (pro-healthy diet index and non-healthy diet index) but does not allow for diet in the context of diet-related diseases to be fully described due to aggregating some foods in one question (e.g., vegetable oils, margarines, and mixes of butter and margarines). Some Polish researchers use two FFQs in one study, which is inconvenient for the respondents and time-consuming for the researchers [14,25,26]. Thus, another FFQ with a more detailed food list is needed.

The study aimed to evaluate the test–retest reproducibility of a non-quantitative food frequency questionnaire (acronym: 62-item FFQ-6) to assess the whole diet and the possibility of identifying dietary patterns in 13–21-year-old females. Young females were selected as a population of interest for several reasons. Adolescent girls, more often than boys, use a variety of diets, e.g., to lose weight or follow a dietary fashion [27,28]. Although girls often make healthier dietary choices than boys [29,30], the overall diet quality decreases with their age [31]. Polish women, due to the traditional division of roles in the family, can strongly influence the dietary habits of their daughters [32,33,34,35]. Therefore, they can influence the diet and health of the next generation.

## 2. Materials and Methods 

### 2.1. Ethical Approval

The study was approved by the Bioethics Committee of the Faculty of Medical Sciences, University of Warmia and Mazury in Olsztyn in June 17, 2010 (resolution no. 20/2010). Informed consent was obtained from adult study participants and from parents/legal guardians of underage girls (<18 years old).

### 2.2. Study Design and Sample Collection

A pilot study was carried out to examine if the questions included in the 62-item FFQ-6 questionnaire were understandable and properly formulated. The main study was carried out in October and November 2012. The questionnaire was completed by each respondent twice with a two-week interval (test and retest). The data were collected by researchers in a face-to-face situation. The researchers described the aim of the study and answering manner in detail to the respondents before starting an interview. The same researcher conducted both the test and the retest with the same respondent (each respondent was assigned an identification number to identify the respondent during the second administration of the questionnaire).

Participants were recruited by contacting students of middle and high schools located in North-Eastern Poland. The intention was to achieve the same proportion of subjects in each age category (13–15, 16–18, and 19–21 years). The inclusion criteria were female gender, age ≥ 13 and ≤21 years and the use of the Polish language in speech. A total of 100 females were recruited. Three girls did not complete at least one questionnaire and they were excluded from the study. The final sample consisted of 97 females aged 13–21 years, including 31 subjects aged 13–15 years, 38 subjects aged 16-18 years, and 28 subjects aged 19–21 years.

### 2.3. A Food Frequency Questionnaire

The 62-item FFQ-6 consists of a list of 62 food items and refers to the usual frequency of food consumption over the last 12 months. The grouping of foods into 62 food items (Table S1) was created based on the authors′ experience and the Food Intake Variety Questionnaire (FIVeQ) with a similar food list whose reproducibility was tested previously [21]. The 62-item FFQ-6 contains two additional questions regarding the consumption frequency of vegetables and fruits in general (questions Q40 and Q29, respectively; Table S4). These two questions can be used by researchers in two ways: (1) to interpret the consumption frequency of vegetables and/or fruits in general (without considering single items), (2) to adjust the consumption frequency of single items of vegetables and fruits, collected with separate questions (Q41–47 and Q30–37, respectively) and then to interpret the consumption frequency of single items of vegetables and fruits in detail. A manual for the adjustment of consumption frequency of single items of vegetables and fruits is included in the Supplementary Materials (Table S4). This study shows the results from crude data, without adjustment for single items of vegetables or fruits.

When answering, to indicate frequency of food consumption, respondents could choose one of six categories (next converted by researcher into daily frequency): never or very rarely (0 times/day), once a month or less (0.025 times/day), several times a month (0.1 times/day), several times a week (0.571 times/day), daily (1 time/day), or a few times a day (2 times/day) [36]. Some food items were aggregated into 25 food items by summing up their daily consumption frequencies (in times/day), and this data set was subjected to further analysis (Table S1).

### 2.4. Statistical Analysis

Categorical variables were presented as a sample percentage (%), and continuous variables as means and standard deviation (SD) [37,38]. The distribution of continuous variables was examined using the Kolmogorov–Smirnov normality test. The reproducibility of a questionnaire was measured by comparing the results of the first interview (test) and the second administration of the questionnaire (retest) in the total sample and by age groups [5]. To comprehensively compare test data and retest data, several statistical measures and tests were used: (i) the Wilcoxon signed rank test for two dependent samples—to verify differences in means of food consumption frequency between the test and the retest; since all continuous variables (as expected) lacked a normal distribution, a non-parametric test was chosen, (ii) Spearman’s correlation coefficient (SCC)—to compare the daily frequency of food consumption (times/day) between test-data and retest-data, (iii) the Fleiss kappa statistic, cross-classification analysis, and chi-square test—to evaluate the agreement of the subject distribution by the same food frequency categories in the test and the retest. The strength of the correlation was interpreted as fair (<0.3), moderate (0.3 to <0.5), good (0.5 to <0.7), or very good (≥0.7). The agreement (measured with the Fleiss' kappa) was interpreted as poor (≤0.2), fair (0.21–0.40), moderate (0.41–0.60), good (0.61–0.80), or very good (≥0.81) [39]. Two-tailed tests were applied and *p*-values < 0.05 were considered as significant.

The principal component analysis (PCA) with varimax normalized rotation was used to identify PCA-driven dietary patterns [40]. For the total sample (*n* = 97), four separate PCA for test data and retest data including a different number of input variables were performed. The input variables were consumption frequencies (in times/day) of: (i) all 60 food items, except for vegetables and fruits in general, (ii) 25 food items after aggregating some items (see Section 2.3). Factor loadings ≥ |0.40| were considered as having a significant contribution toward identifying DPs. Eigenvalues of at least 1.00, scree plot, and variance explained were considered when choosing the best solution. Based on tertile distribution of factor scores of dietary patterns, subjects were divided into three categories within each DP as follows: bottom, middle, and upper tertile. Dietary pattern scores reflecting adherence of each subject to each DP were established. The dietary pattern scores were calculated as a sum of the products of the food consumption frequency and a factor loading for 60 or 25 food items. To evaluate the agreement of DPs identified from data collected with the test and the retest, Spearman correlations for dietary pattern scores were calculated. All analyses were performed with STATISTICA software (version 13.3 PL; StatSoft Inc., Tulsa, OK, USA; StatSoft, Krakow, Poland).

## 3. Results

### 3.1. Mean Frequency of Food Consumption

There were no significant differences in mean frequency of food consumption for most food items reported in the test and the retest in the total sample (57 out of 62) and within age groups (60, 58, and 60 for 13–15, 16–18, and 19–21 years, respectively; Table 1). Significant differences (*p* < 0.05) in mean frequency of food consumption (test vs. retest) were found for olives, wine and cocktails, chocolates, honey, milk and milk beverages—natural in the total sample; for baked confectionery, vegetables, and vegetable-fruit juices in females aged 13–15; for olives, sweetened beverages, honey, and milk and milk beverages—natural in females aged 16–18; and for high-quality cured meats and milk and milk beverages—natural in females aged 19–21.

The Spearman correlations (SCCs) between the frequency of food consumption reported in the test and the retest for food items ranged from 0.24 to 0.86 in the total sample, from 0.09 to 0.84 in females aged 13–15, from 0.41 to 0.91 in females aged 16–18, and from 0.52 to 0.98 in females aged 19–21 (Table 1). The SCC was more than 0.50 (good and very good) for 57 out of 62 food items (92% of total) in the total sample and for 42, 59, and 62 (68%, 95%, and 100%) within the age groups, respectively. 

### 3.2. Categories of Food Consumption Frequency

The kappa statistic (test vs. retest) in the total sample averaged at 0.52 and ranged for food items from 0.32 (root vegetables and others) to 0.72 (spirits), while within age groups, it averaged at 0.41 (0.05–0.66 for food items), 0.53 (0.32–0.71 for food items), and 0.65 (0.37–0.89 for food items), respectively (Table 2). The kappa statistic in the total sample showed moderate agreement (0.41–0.60) for 50 out of 62 food items (81% of total) and good agreement (0.61–0.80) for 8/62 food items (13%). Within age groups, the kappa statistic showed moderate agreement for 26/62 food items (42% of total), 41/62 food items (66%), and 18/62 food items (29%), respectively, and good agreement for 5/62 food items (8% of total), 15/62 food items (24%), and 35/62 food items (57%), respectively. Very good agreement (≥0.81) was found for 7/62 food items (11% of total) in 19–21-year-old females only.

The percentage of subjects classified into the same food frequency category (test vs. retest) in the total sample was on average 68% and ranged for food items from 51% (‘root vegetables and others’) to 89% (venison), while within age groups it averaged at 60%, 68%, and 77%, respectively (Table 2).

### 3.3. Dietary Patterns Identified from 60 Food Items

**Figure 1:**
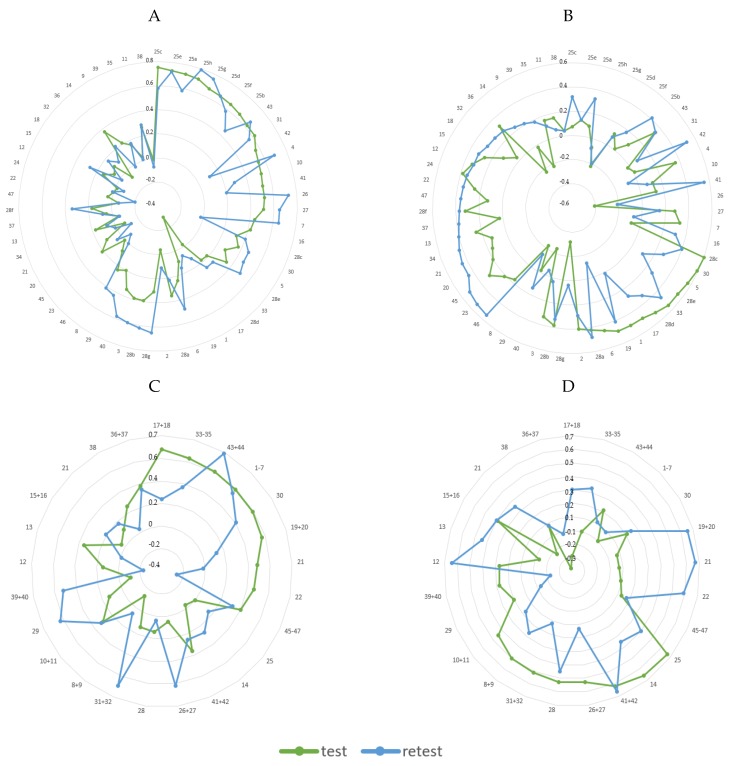
Diagrams with factor loadings of dietary patterns identified in the total sample from a different number of food items in the test and the retest: (**A**) 60-item-DP1, (**B**) 60-item-DP2, (**C**) 25-item-DP1, (**D**) 25-item-DP2. The numbers correspond to food items: 1–sugar; 2–honey; 3–chocolates; 4–sugar confectionery; 5–baked confectionery; 6–ice-creams and custard; 7–savory snacks; 8–milk and milk beverages—natural; 9–cheese curds; 10–milk beverages—sweetened; 11–flavored cheese curds; 12–cheese; 13–eggs and egg dishes; 14–breakfast cereals; 15–whole meal cereals; 16–coarse groats; 17–refined cereals; 18–fine groats; 19–butter; 20–cream; 21–other animal fats; 22–vegetable-based oil; 23–margarine; 24–mayonnaise; 25–all kinds of fruits; 25a–stone fruit; 25b–kiwi and citrus fruit; 25c–tropical fruits; 25d–berries; 25e–bananas; 25f–apples and pears; 25g–avocado; 25h–olives; 26–dried fruit; 27–fruit preserves and fruit condiments; 28–all kinds of vegetables (potatoes not included); 28a–cruciferous vegetables; 28b–yellow-orange vegetables; 28c–leafy green vegetables; 28d–tomatoes; 28e–gourds and squashes; 28f–root vegetables and others; 28g–fresh and tinned legumes; 29–dry and processed pulses; 30–potatoes; 31–nuts and nut spreads; 32–seeds and bran; 33–sausages, bacon, reconstituted meat; 34–high-quality cured meats; 35–offal products; 36–red meat; 37–venison; 38–poultry and rabbit; 39–lean fish; 40–oily fish; 41–fruit juices and nectars; 42–vegetable and vegetable-fruit juices; 43–sweetened beverages; 44–energy drinks; 45–beer; 46–wine and cocktails; 47–spirits.

Considering 60 food items as input variables, two similar dietary patterns (DP1 and DP2) were identified from both test data and retest data (Table S2). The total variance explained for two DPs derived from test data was 23.4%, including 15.9% for the 60-item-DP1 and 7.5% for the 60-item-DP2, and from the retest data it was 25.8%, including 18.0% for the 60-item-DP1 and 7.8% for the 60-item-DP2. Figure 1 presents the factor loadings of dietary patterns identified from different numbers of food items in the test and the retest to visually present the similarities/differences between DPs.

The 60-item-DP1 derived from test-data was positively loaded by the frequency of consumption of tropical fruits (factor loading: 0.75), bananas (0.73); stone fruit (0.72); olives (0.71); avocado (0.67); berries (0.66); apples and pears (0.65); kiwi and citrus fruit (0.64); sweetened beverages (0.62); nuts and nut spread (0.62); vegetable and vegetable-fruit juices (0.56); sugar confectionery (0.54); milk beverages—sweetened (0.53); fruit juices and nectars (0.52); dried fruit (0.52); fruit preserves and fruit condiments (0.51); savory snacks (0.44); and coarse groats (0.42) (Table S2, Figure 1). The 60-item-DP2 derived from test-data was positively loaded by the frequency of consumption of leafy green vegetables (0.60); potatoes (0.59); baked confectionery (0.57); gourds and squashes (0.55); sausages, bacon, reconstituted meat (0.55); tomatoes (0.52); refined cereals (0.49); sugar (0.49); butter (0.49); ice-cream and custard (0.45); cruciferous vegetables (0.42); and honey (0.40) and negatively loaded by the consumption frequency of dried fruit (−0.41).

The Spearman correlation between dietary pattern scores (test vs. retest) in the total sample was 0.84 (within age groups 0.83, 0.81, and 0.78, respectively) for 60-item-DP1 and 0.68 (within age groups 0.24, 0.79, and 0.76, respectively) for the 60-item-DP2 (all *p* < 0.05, except for 60-item-DP2 in females aged 13–15 years; Table 3). The agreement of subject distribution by tertiles of DPs in the total sample was 59% (within age groups 68%, 63%, and 43%, respectively) for the 60-item-DP1, and it was 52% (within age groups 32%, 68%, and 50%, respectively) for the 60-item-DP2 (Table 4). The misclassification to the extreme tertiles in the total sample was 4% (within age groups 0%, 8%, and 4%, respectively) for the 60-item-DP1, and was 7% (within age groups 10%, 5%, and 7%, respectively) for the 60-item-DP2.

### 3.4. Dietary Patterns Identified from 25 Food Items

Considering 25 food items as input variables, two similar dietary patterns (DP1 and DP2) were identified from both test data and retest data (Table S3, Figure 1). The total variance explained for two DPs derived from test data was 26.6%, including 16.1% for the 25-item-DP1 and 10.5% for the 25-item-DP2, and from retest data 28.2%, including 17.7% for the 25-item-DP1 and 10.5% for the 25-item-DP2. 

The 25-item-DP1 derived from test data was positively loaded by the frequency of consumption of refined grain products (factor loading: 0.68), processed meats (0.63); sweetened beverages and energy drinks (0.61); sugar, sweets, and snacks (0.59); potatoes (0.59); butter and cream (0.57); other edible fats (0.48); vegetable oils (0.46); and alcohol (0.40) (Table S3, Figure 1). The 25-item-DP2 derived from test data was positively loaded by the frequency of consumption of fruits (0.67); breakfast cereals (0.66); juices (0.62); dried fruit, fruit preserves, and fruit condiments (0.53); vegetables (0.53); nuts and seeds (0.51); milk, fermented milk drinks, and curd cheese (0.50); and sweetened milk products (0.45).

The Spearman correlation between dietary pattern scores (test vs. retest) in the total sample was 0.76 (within age groups 0.56, 0.82, and 0.89, respectively) for 25-item-DP1, and it was 0.48 (within age groups 0.40, 0.57, and 0.53, respectively) for 25-item-DP2 (all *p* < 0.05; Table 3). The agreement of subject distribution by tertiles of DPs in the total sample was 54% (within age groups 48%, 58%, and 54%, respectively) for the 25-item-DP1, and it was 39% (within age groups 32%, 45%, and 39%, respectively) for the 25-item-DP2 (Table 4). The misclassification to the extreme tertiles in the total sample was 8% (within age groups 10%, 5%, and 11%, respectively) for 25-item-DP1, and it was 14% (within age groups 13%, 13%, and 18%, respectively) for the 25-item-DP2. 

## 4. Discussion

### 4.1. General Reproducibility

It has been found that the reproducibility of the questionnaire was good or very good for most food items regardless of the statistical approach used. In the total sample, the Spearman’s correlation coefficient was on average 0.68, which is considered a good result obtained for FFQ [1]. The same interpretation can be drawn for test–retest reproducibility measured with the kappa statistic (on average 0.52), the percentage of compatible classification into the food frequency category (on average 68%) and the comparison of mean of food consumption frequency (no significant differences for 92% of items). These results are in line with those previously reported. Based on the literature review, Cade et al. [5] stated that correlation coefficients in the range of 0.5–0.7 between test and retest of the FFQs were often reported. For the example, such correlations (0.50–0.70) were found among Brazilian, Danish, Norwegian, and Chinese adolescents [41,42,43,44,45] and American adults (on average 0.70) [46], while a slightly higher correlation (on average 0.78) was noted in Polish young females [22]. In Belgian adolescents, the percentage of compatible classification between test and retest ranged from 37% to 87% [47].

Better reproducibility of the questionnaire was found in older than younger age groups of females (19–21 vs. 16–18 vs. 13–15 years). The Spearman’s correlation coefficient and the kappa statistic tended to be higher in older age groups. For all food items, the highest percentages of compatible classification into the food frequency category were found for the oldest females (19–21 years), while the lowest was for the youngest females (13–15 years). There are a few explanations for the higher reproducibility of the questionnaire in older females (19–21 years). Older females could better identify foods based on its name (given in the questionnaire without a photo gallery or 3D models) and they could better distinguish various types of foods and accurately determine the frequency of consumed food. Older females (as with most adults, in comparison with adolescents) tended to have more stable dietary habits, which are easier to report. Difficulties in precisely assessing dietary intake by adolescent respondents were previously reported [48,49,50]. This was explained by adolescents′ lower knowledge related to food perception and preparation compared to adults and also in rapid changes in dietary habits during adolescence, less eating at home and less supervision by adults. These changes result from a growing sense of independence, peer influence and awareness of social acceptance, greater emotional and financial autonomy, limited time of concentration and attention, and also a lack of interest and motivation to monitor one′s own diet [49,50].

Regardless of the statistical approach used, test–retest reproducibility was the highest for foods consumed occasionally or never (e.g., spirits, olives, venison), and the lowest for foods consumed often (e.g., root vegetables and others, cruciferous vegetables, cheese curds). It can be speculated that some of the foods with lower reproducibility could be consumed as a component of complex dishes (e.g., root vegetables and others in vegetable salad) which could cause difficulties in determining the consumption frequency. A bias in the reported food consumption frequency should be also considered, including overestimation and underestimation. Young people, especially females, may overestimate the consumption of food considered healthy (e.g., vegetables), and to the opposite, can underestimate the consumption of foods considered unhealthy (e.g., fast-foods, soft drinks, sweets, and salty snacks) [51]. In adolescence, requirements for increasing energy supply, concerns related to self-image, and following food fashions may all contribute to poor compliance in dietary reporting [49]. 

### 4.2. Reproducibility of the Identification of Dietary Patterns 

Regardless of the data set used (with 60 or 25 food items), two dietary patterns were identified in the total sample. The first dietary pattern was characterized by the frequent consumption of various types of fruit, sweetened milk products, nuts and seeds, juices, dried fruit, fruit preserves and fruit condiments. This dietary pattern, having a fruit–vegetable–milk profile, can be classified as pro-healthy, although sweet. A similar dietary pattern (the fruit and vegetables) was previously found in a representative sample of Polish females aged 13–21 years [52], in which a positive attitude towards health and natural product interest were revealed. Females chose fruits presumably because of the desire to be healthy and enjoying the good taste of food [53]. Vegetables are also perceived by females as healthy, fashionable, and low-calorie [27,54,55]. Across the world and various subpopulations, healthy or prudent dietary patterns consisting of fruit and vegetables as well as low-fat dairy products, whole grains, legumes, fish and seafood, nuts, and vegetable oils were identified more often [56,57,58]. 

The second dietary pattern was characterized by the frequent consumption of processed meats, refined grain products, potatoes, butter, sugar and sweets. This dietary pattern can be classified as Polish traditional with a westernized profile. Traditional westernized dietary patterns were identified across the world, showing a universal trend toward diet westernization, i.e., a shift from traditional foods towards highly processed, high-fat, high-sugar, and low-fiber foods [13,14,59]. It was discussed that the taste, food availability, and also following food fashions are important factors of food choice, especially for young people [60,61,62]. Karimi-Shahanjarini et al. [61] showed that young females aged 12–15 years were eating unhealthy snacks because of the taste, easy access, and high price of healthy snacks, and the potential risk of disease in the future was not important to them. 

It was found that the dietary patterns identified from retest data were similar to those identified from the test data. This was documented by considering dietary patterns scores in the test and retest. In the total sample, the Spearman’s correlation coefficients were good to very good (0.68–0.84) except one was slightly lower (0.48), and the agreement of subjects’ distribution by tertiles of dietary patterns was acceptable to good (39% to 59%). Based on this, the use of the questionnaire to identify dietary patterns in young Polish females can be recommended. It is possible that the 62-item FFQ-6 can also be used in other European countries with similar dietary habits and food availability, although further investigation is needed. There are limited data regarding the reproducibility of the dietary patterns identified with the FFQs. Such an analysis was previously conducted for American, Spanish, and Japanese adults [46,63,64]. Among American men, two dietary patterns were identified from the 131-item FFQ [46]. The correlations between first and second (1 year apart) administration of the FFQ were good: 0.70 for the Prudent pattern and 0.67 for the Western pattern. In Japanese adults, the reproducibility of dietary patterns was reported based on a systematic review of PCA-derived dietary patterns [64]. The reproducibility of dietary patterns was assessed using a congruence coefficient (CC). When high quality data, i.e., coming from a validated FFQ or multiple-day dietary records and sample size ≥ 200, were included, the median CC was high for Healthy pattern (0.89), Prudent pattern (0.86), and Japanese pattern (0.80), while it was low for the Traditional pattern (0.59), Western pattern (0.44), and Traditional Japanese pattern (0.31). This systematic review has shown that there are some dietary patterns that are relatively reproducible in different populations in a given country. The reproducibility of data-driven dietary patterns was assessed in different Spanish samples extracted from similar populations [63] using congruence coefficients similarly to the Japanese study [64]. The median of the CC was 0.90 for Western pattern, 0.77 for the Mediterranean pattern, and 0.76 for Prudent pattern. Due to a lack of similar studies covering respondents at the same age (13–21 years), and taking into account that reporting of food consumption is more biased in adolescents than adults [49], the current findings (i.e., Spearman’s correlation 0.48–0.84) cannot be directly compared with those cited above. However, it can be speculated that reproducibility of pro-healthy dietary patterns (e.g., Prudent) is better than non-healthy dietary patterns (e.g., Western).

It is difficult to comprehensively discuss the reproducibility of dietary patterns identified across the age groups (13–15 vs. 16–18 vs. 19–21 years) due to the small number of subjects (31 vs. 38 vs. 28, respectively). However, showing great caution in reasoning, it can be suggested that better reproducibility of dietary patterns identification was found in older than younger age groups of females. A possible explanation was discussed in Section 4.1.

### 4.3. Strengths and Limitations

The main strength of the study is applying several methods of statistical analysis as recommended, all suitable for an evaluation of the FFQs’ reproducibility [5,65]. Such an approach—the application of multiple statistical tests—allows for gaining comprehensive insights, reduces the chance of an over-interpretation of research findings, strengthens the conclusions, and increases the possibility of comparing these results with others. Secondly, using two data sets, i.e., with 60 and 25 food items, provided the possibility of using the questionnaire to identify dietary patterns from non-aggregated and aggregated food items and, indirectly, the usefulness of the questionnaire in terms of processing the dietary data obtained. This indicates the possibilities offered by this questionnaire and may be an inspiration for less advanced researchers. To facilitate future use of this questionnaire by other researchers, the manual (Table S4) is attached.

The limitation of the study is the relatively small number of subjects (97). The findings related to the age groups should be particularly interpreted with caution (as tendency) due to the small number of respondents (31, 38, and 28). However, in FFQ validation studies, similarly numbered samples (in total 48–90) were previously reported in adolescents from Denmark [43], Brazil [41], New Zealand [11], and Norway [44], and also in young Polish females [17,20]. Furthermore, similarly numbered sub-samples (40–66) were analyzed across the age, sex, or ethnicity in children and adolescents [66] and adults [39,67]. When a principal component analysis (PCA) was performed from 60 food items, the subject-to-item ratio was low (1.6:1, i.e., 97 subjects to 60 items), but for 25 food items it can be considered as sufficient (3.9:1, i.e., 97 subjects to 25 items) because it was slightly below the lower border of the recommended range of the subject-to-item ratio [68,69]. Regardless of the limitations in the PCA, it should be emphasized that the PCA was performed to assess repeatability in identifying dietary patterns, and not to interpret them comprehensively. Since only one gender group of Polish residents was selected with a narrow age range (13–21 years), these findings cannot be applied to people of different age or gender. Even so, it may be supposed that similar relations can be found in other European females of a similar age. Since dietary interviews conducted in adults are less burdened by measurement errors compared to adolescents [49], it can be speculated that this questionnaire can be used in adults of both sexes as they provide relatively reliable dietary data. In the present study, the reproducibility was assessed for a questionnaire administrated by trained interviewers, so it can be assumed that the reproducibility will be lower for a self-administered questionnaire. Previously, the better reproducibility of the interviewer-administered KomPAN® questionnaire than its self-administered version was revealed in Polish adolescents and adults [24]. 

## 5. Conclusions

The test–retest reproducibility of the 62-item FFQ-6 was good or very good for most food items, with a tendency to be higher in older age groups of females under study. Due to the acceptable-to-good reproducibility of identification of dietary patterns derived using the principal component analysis, the use of 62-item FFQ-6 to describe the overall diet of Polish young females can be recommended.

## Figures and Tables

**Table 1:** Comparison of food consumption frequency (times/day) in the test and the retest by age groups (mean and standard deviation).

Food Items ^1^	Total Sample (*n* = 97)				13–15 Years (*n* = 31)				16–18 Years (*n* = 38)				19–21 Years (*n* = 28)			
	Test	Retest	*p*	SCC	Test	Retest	*p*	SCC	Test	Retest	*p*	SCC	Test	Retest	*p*	SCC
Olives	0.08 ± 0.31	0.05 ± 0.23	0.046	0.86 *	0.11 ± 0.39	0.08 ± 0.36	0.893	0.75 *	0.11 ± 0.35	0.06 ± 0.18	0.028	0.89 *	0.02 ± 0.04	0.02 ± 0.04	0.180	0.94 *
Beer	0.09 ± 0.18	0.12 ± 0.22	0.147	0.85 *	0.05 ± 0.14	0.08 ± 0.22	0.529	0.71 *	0.13 ± 0.22	0.15 ± 0.24	0.575	0.89 *	0.10 ± 0.14	0.12 ± 0.17	0.109	0.92 *
Wine and cocktails	0.04 ± 0.09	0.07 ± 0.23	0.013	0.83 *	0.00 ± 0.02	0.07 ± 0.36	0.273	0.61 *	0.06 ± 0.13	0.09 ± 0.17	0.051	0.83 *	0.04 ± 0.04	0.06 ± 0.11	0.285	0.94 *
Spirits	0.04 ± 0.09	0.05 ± 0.14	0.209	0.83 *	0.01 ± 0.03	0.06 ± 0.20	0.109	0.74 *	0.06 ± 0.13	0.05 ± 0.10	0.953	0.78 *	0.04 ± 0.04	0.05 ± 0.11	0.655	0.98 *
Sugar confectionery	0.38 ± 0.45	0.41 ± 0.45	0.449	0.82 *	0.42 ± 0.41	0.52 ± 0.48	0.080	0.73 *	0.35 ± 0.43	0.34 ± 0.31	0.753	0.81 *	0.38 ± 0.53	0.40 ± 0.56	0.735	0.91 *
Sugar	0.95 ± 0.71	1.00 ± 0.73	0.155	0.81 *	0.79 ± 0.62	0.84 ± 0.65	0.826	0.56 *	0.89 ± 0.70	0.94 ± 0.71	0.477	0.89 *	1.22 ± 0.77	1.26 ± 0.78	0.673	0.83 *
Dried fruit	0.09 ± 0.20	0.08 ± 0.24	0.381	0.81 *	0.14 ± 0.28	0.17 ± 0.41	0.730	0.75 *	0.06 ± 0.13	0.03 ± 0.03	0.141	0.78 *	0.06 ± 0.15	0.03 ± 0.04	0.225	0.90 *
High quality cured meats	0.51 ± 0.45	0.54 ± 0.45	0.122	0.80 *	0.51 ± 0.50	0.48 ± 0.43	0.814	0.71 *	0.59 ± 0.46	0.63 ± 0.50	0.333	0.83 *	0.40 ± 0.34	0.48 ± 0.38	0.043	0.90 *
Other animal fats	0.01 ± 0.02	0.02 ± 0.06	0.117	0.80 *	0.01 ± 0.02	0.01 ± 0.03	0.584	0.74 *	0.01 ± 0.02	0.03 ± 0.09	0.075	0.79 *	0.01 ± 0.02	0.01 ± 0.02	1.000	0.86 *
Tomatoes	0.77 ± 0.59	0.70 ± 0.54	0.084	0.79 *	0.58 ± 0.45	0.55 ± 0.35	0.834	0.73 *	0.77 ± 0.60	0.64 ± 0.58	0.069	0.69 *	0.97 ± 0.64	0.94 ± 0.60	0.593	0.95 *
Poultry and rabbit	0.34 ± 0.36	0.30 ± 0.29	0.163	0.79 *	0.39 ± 0.36	0.36 ± 0.35	0.507	0.77 *	0.35 ± 0.41	0.28 ± 0.27	0.308	0.80 *	0.27 ± 0.25	0.25 ± 0.25	0.361	0.81 *
Energy drinks	0.12 ± 0.20	0.15 ± 0.25	0.074	0.79 *	0.15 ± 0.23	0.17 ± 0.27	0.594	0.63 *	0.13 ± 0.21	0.18 ± 0.27	0.173	0.85 *	0.07 ± 0.11	0.11 ± 0.17	0.106	0.86 *
Chocolates	0.68 ± 0.57	0.61 ± 0.49	0.032	0.78 *	0.78 ± 0.62	0.66 ± 0.46	0.266	0.57 *	0.71 ± 0.55	0.66 ± 0.44	0.424	0.79 *	0.55 ± 0.53	0.50 ± 0.57	0.080	0.91 *
Savory snacks	0.34 ± 0.39	0.34 ± 0.43	1.000	0.78 *	0.35 ± 0.43	0.37 ± 0.51	0.683	0.68 *	0.35 ± 0.33	0.41 ± 0.43	0.196	0.82 *	0.30 ± 0.44	0.22 ± 0.28	0.173	0.82 *
Mayonnaise	0.21 ± 0.31	0.19 ± 0.30	0.453	0.78 *	0.14 ± 0.20	0.14 ± 0.20	0.838	0.75 *	0.27 ± 0.41	0.25 ± 0.40	0.463	0.72 *	0.19 ± 0.24	0.17 ± 0.22	0.735	0.86 *
Butter	0.98 ± 0.71	0.98 ± 0.67	0.911	0.77 *	0.88 ± 0.65	0.83 ± 0.52	0.367	0.69 *	1.04 ± 0.75	1.13 ± 0.72	0.255	0.76 *	0.99 ± 0.74	0.92 ± 0.72	0.263	0.92 *
Fruit juices and nectars	0.63 ± 0.55	0.57 ± 0.53	0.267	0.77 *	0.70 ± 0.62	0.51 ± 0.52	0.091	0.64 *	0.72 ± 0.51	0.70 ± 0.52	0.636	0.80 *	0.42 ± 0.45	0.46 ± 0.53	0.441	0.90 *
Kiwi and citrus fruit	0.44 ± 0.47	0.46 ± 0.47	0.478	0.77 *	0.57 ± 0.58	0.56 ± 0.51	0.683	0.72 *	0.46 ± 0.43	0.50 ± 0.50	0.861	0.69 *	0.27 ± 0.32	0.29 ± 0.32	0.361	0.92 *
Leafy green vegetables	0.36 ± 0.38	0.37 ± 0.37	0.699	0.77 *	0.22 ± 0.27	0.28 ± 0.31	0.328	0.61 *	0.37 ± 0.37	0.39 ± 0.34	0.450	0.76 *	0.50 ± 0.46	0.43 ± 0.46	0.068	0.94 *
Sweetened beverages	0.33 ± 0.38	0.37 ± 0.45	0.255	0.77 *	0.35 ± 0.42	0.41 ± 0.51	0.638	0.63 *	0.36 ± 0.42	0.45 ± 0.50	0.018	0.89 *	0.26 ± 0.28	0.22 ± 0.23	0.463	0.77 *
Gourds and squashes	0.58 ± 0.56	0.55 ± 0.51	0.451	0.76 *	0.51 ± 0.45	0.53 ± 0.45	0.780	0.67 ^*^	0.55 ± 0.62	0.45 ± 0.50	0.130	0.71 *	0.69 ± 0.59	0.69 ± 0.57	0.686	0.92 *
Cheese	0.68 ± 0.48	0.64 ± 0.48	0.243	0.75 *	0.55 ± 0.46	0.52 ± 0.45	0.972	0.84 *	0.68 ± 0.47	0.64 ± 0.47	0.534	0.62 *	0.84 ± 0.49	0.78 ± 0.49	0.141	0.81 *
Margarine	0.26 ± 0.44	0.27 ± 0.44	0.287	0.74 *	0.14 ± 0.28	0.16 ± 0.25	0.163	0.48 *	0.33 ± 0.50	0.40 ± 0.60	0.477	0.89 *	0.30 ± 0.47	0.23 ± 0.33	0.529	0.82 *
All kinds of fruits	0.89 ± 0.54	0.88 ± 0.56	0.936	0.73 *	1.02 ± 0.60	0.98 ± 0.57	0.834	0.61 *	0.88 ± 0.54	0.86 ± 0.59	0.889	0.73 *	0.77 ± 0.47	0.79 ± 0.52	0.465	0.87 *
Sausages, bacon, reconstituted meat	0.45 ± 0.47	0.42 ± 0.47	0.194	0.73 *	0.37 ± 0.32	0.30 ± 0.36	0.133	0.52 *	0.44 ± 0.50	0.46 ± 0.51	0.807	0.73 *	0.56 ± 0.55	0.52 ± 0.52	0.263	0.91 *
Eggs and egg dishes	0.22 ± 0.25	0.26 ± 0.27	0.099	0.73 *	0.20 ± 0.22	0.28 ± 0.30	0.142	0.75 *	0.24 ± 0.25	0.32 ± 0.29	0.074	0.70 *	0.22 ± 0.28	0.17 ± 0.19	0.281	0.76 *
Oily fish	0.06 ± 0.12	0.06 ± 0.10	0.844	0.73 *	0.07 ± 0.14	0.09 ± 0.17	0.209	0.78 *	0.05 ± 0.09	0.04 ± 0.04	0.327	0.74 *	0.07 ± 0.15	0.04 ± 0.04	0.953	0.70 *
Lean fish	0.10 ± 0.18	0.08 ± 0.17	0.269	0.72 *	0.10 ± 0.22	0.12 ± 0.26	0.583	0.60 *	0.10 ± 0.17	0.07 ± 0.13	0.154	0.80 *	0.10 ± 0.17	0.05 ± 0.04	0.142	0.81 *
Fruit preserves and fruit condiments	0.30 ± 0.37	0.33 ± 0.42	0.279	0.71 *	0.30 ± 0.33	0.32 ± 0.46	0.931	0.59 *	0.25 ± 0.37	0.35 ± 0.42	0.055	0.76 *	0.37 ± 0.41	0.34 ± 0.40	0.674	0.77 *
Honey	0.05 ± 0.10	0.10 ± 0.19	0.003	0.71 *	0.02 ± 0.03	0.07 ± 0.20	0.142	0.36 *	0.05 ± 0.10	0.12 ± 0.20	0.033	0.85 *	0.07 ± 0.15	0.10 ± 0.17	0.123	0.80 *
Bananas	0.42 ± 0.43	0.41 ± 0.38	0.880	0.70 *	0.56 ± 0.53	0.50 ± 0.42	0.824	0.63 *	0.31 ± 0.39	0.33 ± 0.38	0.351	0.70 *	0.41 ± 0.33	0.41 ± 0.34	0.933	0.75 *
Refined cereals	0.92 ± 0.59	0.98 ± 0.63	0.237	0.69 *	0.91 ± 0.61	0.98 ± 0.61	0.422	0.50 *	0.86 ± 0.59	0.86 ± 0.61	0.826	0.81 *	1.00 ± 0.57	1.13 ± 0.67	0.182	0.67 *
Vegetable-based oil	0.41 ± 0.43	0.37 ± 0.44	0.190	0.69 *	0.36 ± 0.34	0.27 ± 0.30	0.163	0.46 *	0.50 ± 0.56	0.48 ± 0.60	0.784	0.82 *	0.34 ± 0.28	0.33 ± 0.28	0.673	0.73 *
Breakfast cereals	0.34 ± 0.36	0.34 ± 0.39	0.784	0.69 *	0.43 ± 0.40	0.40 ± 0.47	0.663	0.67 *	0.35 ± 0.38	0.36 ± 0.36	0.809	0.68 *	0.22 ± 0.27	0.24 ± 0.30	0.735	0.75 *
Whole meal cereals	0.61 ± 0.64	0.64 ± 0.61	0.826	0.68 *	0.57 ± 0.63	0.73 ± 0.68	0.438	0.38 *	0.66 ± 0.70	0.68 ± 0.66	0.610	0.91 *	0.60 ± 0.57	0.48 ± 0.40	0.445	0.69 *
Baked confectionery	0.36 ± 0.45	0.37 ± 0.41	0.466	0.68 *	0.28 ± 0.29	0.39 ± 0.38	0.037	0.59 *	0.30 ± 0.33	0.32 ± 0.31	0.778	0.63 *	0.51 ± 0.67	0.43 ± 0.55	0.208	0.85 *
Nuts and nut spreads	0.21 ± 0.32	0.20 ± 0.35	0.357	0.68 *	0.28 ± 0.30	0.29 ± 0.43	0.824	0.61 *	0.27 ± 0.40	0.24 ± 0.38	0.535	0.55 *	0.06 ± 0.04	0.05 ± 0.04	0.173	0.80 *
Berries	0.35 ± 0.45	0.29 ± 0.37	0.178	0.67 *	0.45 ± 0.60	0.33 ± 0.42	0.334	0.42 *	0.36 ± 0.36	0.32 ± 0.36	0.366	0.75 *	0.25 ± 0.33	0.22 ± 0.32	0.834	0.78 *
Avocado	0.03 ± 0.21	0.04 ± 0.23	0.959	0.67 *	0.07 ± 0.36	0.10 ± 0.40	0.753	0.44 *	0.02 ± 0.09	0.02 ± 0.09	0.800	0.73 *	0.01 ± 0.02	0.01 ± 0.02	0.789	0.86 *
Venison	0.00 ± 0.02	0.01 ± 0.02	0.197	0.67 *	0.00 ± 0.02	0.01 ± 0.03	0.109	0.75 *	0.01 ± 0.02	0.01 ± 0.01	1.000	0.58 *	0.00 ± 0.01	0.00 ± 0.01	NA	0.80 *
Potatoes	0.79 ± 0.46	0.78 ± 0.47	0.952	0.66 *	0.78 ± 0.43	0.83 ± 0.47	0.478	0.51 *	0.84 ± 0.45	0.77 ± 0.46	0.295	0.57 *	0.74 ± 0.50	0.75 ± 0.49	0.675	0.89 *
Apples and pears	0.68 ± 0.46	0.66 ± 0.43	0.487	0.66 *	0.86 ± 0.51	0.77 ± 0.43	0.249	0.56 *	0.56 ± 0.48	0.62 ± 0.48	0.505	0.57 *	0.63 ± 0.31	0.58 ± 0.34	0.173	0.81 *
Cheese curds	0.29 ± 0.35	0.27 ± 0.27	0.887	0.66 *	0.27 ± 0.33	0.22 ± 0.29	0.328	0.59 *	0.29 ± 0.32	0.32 ± 0.25	0.267	0.65 *	0.31 ± 0.43	0.25 ± 0.27	0.415	0.80 *
Tropical fruits	0.15 ± 0.29	0.20 ± 0.37	0.202	0.65 *	0.19 ± 0.41	0.27 ± 0.53	0.397	0.50 *	0.16 ± 0.24	0.21 ± 0.30	0.327	0.77 *	0.09 ± 0.17	0.10 ± 0.17	0.534	0.62 *
Flavored cheese curds	0.13 ± 0.22	0.13 ± 0.28	0.311	0.65 *	0.12 ± 0.23	0.18 ± 0.42	1.000	0.48 *	0.17 ± 0.25	0.13 ± 0.20	0.507	0.76 *	0.09 ± 0.14	0.08 ± 0.14	0.260	0.70 *
Fresh and tinned legumes	0.13 ± 0.19	0.14 ± 0.28	0.531	0.63 *	0.16 ± 0.21	0.21 ± 0.41	0.875	0.40 *	0.14 ± 0.22	0.11 ± 0.19	0.286	0.70 *	0.10 ± 0.14	0.11 ± 0.20	0.715	0.86 *
Red meat	0.10 ± 0.17	0.10 ± 0.16	0.915	0.63 *	0.05 ± 0.10	0.09 ± 0.17	0.108	0.70 *	0.10 ± 0.17	0.09 ± 0.15	0.551	0.56 *	0.15 ± 0.20	0.12 ± 0.17	0.173	0.59 *
Cream	0.24 ± 0.33	0.24 ± 0.33	0.928	0.62 *	0.24 ± 0.28	0.22 ± 0.25	0.925	0.35	0.26 ± 0.41	0.29 ± 0.42	0.601	0.65 *	0.21 ± 0.26	0.19 ± 0.25	0.590	0.91 *
Stone fruit	0.45 ± 0.49	0.38 ± 0.42	0.052	0.61 *	0.57 ± 0.65	0.43 ± 0.46	0.246	0.40 *	0.45 ± 0.42	0.43 ± 0.45	0.363	0.64 *	0.32 ± 0.33	0.25 ± 0.30	0.128	0.84 *
Milk beverages—sweetened	0.51 ± 0.44	0.51 ± 0.39	0.622	0.60 *	0.58 ± 0.51	0.48 ± 0.41	0.449	0.29	0.55 ± 0.45	0.58 ± 0.41	0.334	0.71 *	0.37 ± 0.32	0.44 ± 0.33	0.075	0.86 *
Ice-cream and custard	0.24 ± 0.33	0.21 ± 0.36	0.076	0.60 *	0.27 ± 0.39	0.21 ± 0.40	0.142	0.35	0.22 ± 0.31	0.17 ± 0.25	0.244	0.71 *	0.22 ± 0.30	0.25 ± 0.43	0.625	0.72 *
Seeds and bran	0.15 ± 0.26	0.17 ± 0.32	0.637	0.59 *	0.20 ± 0.29	0.19 ± 0.29	0.542	0.26	0.20 ± 0.31	0.26 ± 0.42	0.328	0.78 *	0.03 ± 0.03	0.02 ± 0.02	0.124	0.64 *
Milk and milk beverages—natural	0.61 ± 0.47	0.52 ± 0.46	0.015	0.58 *	0.59 ± 0.53	0.62 ± 0.65	0.811	0.52 *	0.62 ± 0.48	0.47 ± 0.36	0.041	0.58 *	0.62 ± 0.40	0.47 ± 0.30	0.018	0.76 *
Vegetable and vegetable-fruit juices	0.28 ± 0.41	0.22 ± 0.31	0.075	0.58 *	0.35 ± 0.51	0.15 ± 0.21	0.005	0.58 *	0.29 ± 0.39	0.33 ± 0.41	0.642	0.52 *	0.18 ± 0.26	0.13 ± 0.19	0.263	0.74 *
Offal products	0.05 ± 0.11	0.07 ± 0.23	0.483	0.54 *	0.06 ± 0.14	0.12 ± 0.38	0.784	0.52 *	0.03 ± 0.04	0.06 ± 0.13	0.826	0.44 *	0.07 ± 0.15	0.04 ± 0.11	0.142	0.74 *
Fine groats	0.15 ± 0.21	0.13 ± 0.16	0.474	0.51 *	0.16 ± 0.23	0.14 ± 0.17	0.894	0.36 *	0.14 ± 0.19	0.12 ± 0.16	0.878	0.63 *	0.16 ± 0.20	0.11 ± 0.13	0.333	0.52 *
All kinds of vegetables (potatoes not included)	0.69 ± 0.47	0.71 ± 0.46	0.837	0.50 *	0.68 ± 0.54	0.73 ± 0.56	0.650	0.35	0.74 ± 0.53	0.71 ± 0.49	0.496	0.56 *	0.64 ± 0.30	0.69 ± 0.29	0.273	0.70 *
Coarse groats	0.09 ± 0.16	0.09 ± 0.13	0.911	0.48 *	0.17 ± 0.27	0.11 ± 0.16	0.712	0.36 *	0.06 ± 0.04	0.10 ± 0.15	0.155	0.41 *	0.06 ± 0.04	0.05 ± 0.04	0.059	0.70 *
Root vegetables and others	0.39 ± 0.35	0.37 ± 0.37	0.335	0.45 *	0.34 ± 0.32	0.33 ± 0.31	0.862	0.09	0.39 ± 0.36	0.33 ± 0.42	0.124	0.53 *	0.46 ± 0.36	0.46 ± 0.36	0.878	0.74 *
Dry and processed pulses	0.07 ± 0.10	0.08 ± 0.15	0.685	0.43 *	0.07 ± 0.10	0.10 ± 0.16	0.679	0.27	0.07 ± 0.09	0.09 ± 0.18	0.514	0.48 *	0.06 ± 0.11	0.04 ± 0.04	0.612	0.58 *
Yellow-orange vegetables	0.50 ± 0.42	0.45 ± 0.36	0.204	0.33 *	0.50 ± 0.48	0.43 ± 0.44	0.334	0.39 *	0.53 ± 0.43	0.48 ± 0.32	0.799	0.63 *	0.46 ± 0.32	0.43 ± 0.31	0.499	0.76 *
Cruciferous vegetables	0.29 ± 0.34	0.28 ± 0.28	0.981	0.24 *	0.20 ± 0.27	0.20 ± 0.23	0.784	0.48 *	0.35 ± 0.41	0.32 ± 0.31	0.646	0.81 *	0.31 ± 0.30	0.31 ± 0.27	0.787	0.72 *
Mean for all food items	-	-		0.68	-	-		0.56	-	-		0.72	-	-		0.80

^1^ Sorted by SCC values for the total sample; *n*—sample size; SCC—Spearman’s correlation coefficient: * *p* < 0.05; *p*–significance level of Wilcoxon’s test (for two dependent samples) for differences in means of food consumption frequency (times/day) between the test and the retest; NA–statistical analysis was not performed in this age group due to sample distribution (not enough respondents in a category).

**Table 2:** Classification agreement and misclassification of food consumption frequency in the test and the retest by age groups (%).

Food Items ^1^	Total Sample (*n* = 97)				13–15 Years (*n* = 31)				16–18 Years (*n* = 38)				19–21 Years (*n* = 28)			
	Compatible	Non-Compatible		*k*	Compatible	Non-Compatible		*k*	Compatible	Non-Compatible		*k*	Compatible	Non-Compatible		*k*
		±1	±2 or More			±1	±2 or More			±1	±2 or More			±1	±2 or More	
Venison	89	10	1	0.56	90	6	3	0.58	82	18	0	0.50	96	4	0	0.78
Other animal fats	88	10	2	0.64	87	10	3	0.54	84	13	3	0.53	93	7	0	0.85
Olives	87	11	2	0.70	84	10	6	0.65	84	16	0	0.71	93	7	0	0.86
Spirits	86	10	4	0.72	90	3	6	0.66	76	18	5	0.63	93	7	0	0.89
Avocado	84	15	1	0.54	81	16	3	0.42	82	18	0	0.61	89	11	0	0.52
Wine and cocktails	84	12	4	0.71	87	6	6	0.50	76	18	5	0.66	89	11	0	0.84
Beer	80	18	2	0.70	81	13	6	0.62	74	26	0	0.64	89	11	0	0.83
All kinds of fruits	74	22	4	0.61	74	19	6	0.63	66	32	3	0.52	86	11	4	0.79
Energy drinks	74	19	7	0.63	65	26	10	0.51	76	16	8	0.68	82	14	4	0.74
Eggs and egg dishes	73	24	3	0.56	65	32	3	0.48	74	24	3	0.59	82	14	4	0.64
Tomatoes	73	18	9	0.62	74	16	10	0.63	61	24	16	0.49	89	11	0	0.85
Chocolates	72	23	5	0.60	65	23	13	0.48	71	26	3	0.60	82	18	0	0.76
Kiwi and citrus fruit	72	23	5	0.59	68	23	10	0.56	66	29	5	0.53	86	14	0	0.77
Dried fruit	72	23	5	0.57	55	32	13	0.37	79	18	3	0.67	82	18	0	0.73
Cruciferous vegetables	72	24	4	0.55	61	29	10	0.41	74	26	0	0.60	82	14	4	0.70
Poultry and rabbit	72	25	3	0.59	58	39	3	0.44	74	24	3	0.62	86	11	4	0.79
Sugar confectionery	71	26	3	0.58	74	19	6	0.60	66	32	3	0.53	75	25	0	0.67
Savory snacks	71	26	3	0.58	68	29	3	0.55	68	29	3	0.57	79	18	4	0.67
Leafy green vegetables	71	24	5	0.60	58	35	6	0.43	71	21	8	0.60	86	14	0	0.81
Lean fish	71	23	6	0.57	61	26	13	0.46	74	24	3	0.63	79	18	4	0.67
Bananas	70	26	4	0.54	65	29	6	0.49	71	26	3	0.56	75	21	4	0.62
Fresh and tinned legumes	70	22	8	0.54	55	29	16	0.37	71	24	5	0.56	86	11	4	0.76
High quality cured meats	70	27	3	0.56	61	32	6	0.46	74	24	3	0.61	75	25	0	0.65
Honey	69	25	6	0.53	65	23	13	0.39	71	26	3	0.59	71	25	4	0.60
Mayonnaise	69	27	4	0.56	68	29	3	0.54	66	26	8	0.53	75	25	0	0.64
Fine groats	68	23	9	0.46	65	16	19	0.42	74	21	5	0.58	64	32	4	0.38
Apples and pears	68	26	6	0.51	58	39	3	0.34	68	21	11	0.56	79	18	4	0.66
Sugar	67	27	6	0.54	55	29	16	0.39	71	26	3	0.61	75	25	0	0.63
Baked confectionery	67	26	7	0.50	68	19	13	0.46	63	29	8	0.47	71	29	0	0.59
Cheese	67	25	8	0.53	58	29	13	0.44	71	18	11	0.59	71	29	0	0.58
Coarse groats	67	24	9	0.47	52	35	13	0.29	71	18	11	0.51	79	18	4	0.64
Butter	67	23	10	0.53	61	23	16	0.36	68	21	11	0.57	71	25	4	0.63
Yellow-orange vegetables	67	25	8	0.49	52	29	19	0.30	74	21	5	0.61	75	25	0	0.60
Nuts and nut spreads	66	28	6	0.50	65	23	13	0.48	58	37	5	0.40	79	21	0	0.66
Red meat	66	26	8	0.51	58	39	3	0.40	63	24	13	0.48	79	14	7	0.68
Oily fish	66	33	1	0.48	61	39	0	0.45	68	32	0	0.51	68	29	4	0.51
All kinds of vegetables (potatoes not included)	65	28	7	0.47	52	35	13	0.34	61	34	5	0.42	86	11	4	0.76
Potatoes	65	32	3	0.46	52	45	3	0.23	66	29	5	0.47	79	21	0	0.69
Sausages, bacon, reconstituted meat	65	26	9	0.54	58	26	16	0.45	66	26	8	0.56	71	25	4	0.64
Vegetable-based oil	64	32	4	0.48	48	42	10	0.29	68	29	3	0.58	75	25	0	0.60
Margarine	64	27	9	0.52	48	32	19	0.31	71	26	3	0.63	71	21	7	0.62
Gourds and squashes	64	27	9	0.51	58	29	13	0.43	55	32	13	0.43	82	18	0	0.76
Sweetened beverages	64	31	5	0.50	55	35	10	0.38	61	37	3	0.49	79	18	4	0.67
Flavored cheese curds	63	28	9	0.48	55	29	16	0.39	66	29	5	0.54	68	25	7	0.54
Whole meal cereals	63	23	14	0.52	48	23	29	0.36	74	21	5	0.68	64	25	11	0.53
Milk and milk beverages—natural	62	25	13	0.46	42	39	19	0.26	68	18	13	0.57	75	18	7	0.58
Cream	62	28	10	0.47	55	26	19	0.38	53	37	11	0.39	82	18	0	0.74
Tropical fruits	62	30	8	0.43	55	29	16	0.37	68	24	8	0.54	61	39	0	0.37
Offal products	62	27	11	0.42	61	26	13	0.42	63	24	13	0.44	61	32	7	0.41
Fruit juices and nectars	62	34	4	0.48	65	26	10	0.52	55	42	3	0.39	68	32	0	0.56
Milk beverages—sweetened	61	28	11	0.44	45	26	29	0.22	61	34	5	0.45	79	21	0	0.68
Refined cereals	61	35	4	0.44	58	35	6	0.39	63	34	3	0.50	61	36	4	0.43
Dry and processed pulses	61	27	12	0.41	48	35	16	0.24	61	26	13	0.41	75	18	7	0.58
Seeds and bran	61	29	10	0.44	42	32	26	0.23	71	24	5	0.61	68	32	0	0.42
Stone fruit	60	33	7	0.42	45	39	16	0.27	61	34	5	0.42	75	25	0	0.64
Berries	60	31	9	0.45	52	29	19	0.32	58	37	5	0.44	71	25	4	0.61
Fruit preserves and fruit condiments	60	31	9	0.46	42	45	13	0.21	66	26	8	0.54	71	21	7	0.61
Vegetable and vegetable-fruit juices	60	23	18	0.45	52	26	23	0.34	58	24	18	0.44	71	18	11	0.61
Cheese curds	58	37	5	0.40	58	32	10	0.44	53	42	5	0.32	64	36	0	0.47
Ice-creams and custard	56	41	3	0.35	45	45	10	0.15	61	39	0	0.40	61	39	0	0.45
Breakfast cereals	55	36	9	0.39	42	45	13	0.25	50	42	8	0.32	75	18	7	0.65
Root vegetables and others	51	32	18	0.32	32	35	32	0.05	55	26	18	0.41	64	36	0	0.47
Mean for all food items	68	25	7	0.52	60	28	12	0.41	68	26	6	0.53	77	21	3	0.65

^1^ Sorted by compatible classification (%) for the total sample; *k*–the Fleiss’ kappa.

**Table 3:** Spearman correlations for scores of dietary patterns (DP) identified in the total sample in the test and the retest by age group.

Age Group	DP1		*p*	DP2		*p*
	60-Item-DP1	25-Item-DP1		60-Item-DP2	25-Item-DP2	
Total sample (*n* = 97)	0.84 *	0.76 *	0.1247	0.68 *	0.48 *	0.0372
13-15 years (*n* = 31)	0.83 *	0.56 *	0.0422	0.24	0.40 *	0.5060
16-18 years (*n* = 38)	0.81 *	0.82 *	0.9012	0.79 *	0.57 *	0.0804
19-21 years (*n* = 28)	0.78 *	0.89 *	0.1889	0.76 *	0.53 *	0.1571

*n*—sample size; significance level of Spearman's rank correlation: * *p* < 0.05; *p*—significance value for comparison of Spearman correlations between DPs identified from different number of food items (60 or 25).

**Table 4:** Agreement of subjects’ distribution by tertiles of dietary patterns (DPs) identified in the total sample in the test and the retest by age group (%).

Agreement of Subjects’ Distribution	DP1		DP2	
	60-Item-DP1	25-Item-DP1	60-Item-DP2	25-Item-DP2
Total sample (*n* = 97)				
Compatible	59	54 ^a^	52	39 ^a^
Non-compatible ± 1 category	37	38	41	46
Non-compatible ± 2 category	4	8	7	14
13–15 years (*n* = 31)				
Compatible	68 ^b^	48	32 ^b^	32
Non-compatible ±1 category	32 ^c^	42	58 ^c^	55
Non-compatible ±2 category	0	10	10	13
16–18 years (*n* = 38)				
Compatible	63	58	68	45
Non-compatible ±1 category	29	37	26	42
Non-compatible ±2 category	8	5	5	13
19–21 years (*n* = 28)				
Compatible	43	54	50	39
Non-compatible ±1 category	54	36	43	43
Non-compatible ±2 category	4	11	7	18

*n*—sample size; the same letters ^(a-a, b-b, c-c)^ indicate statistically significant difference (*p* < 0.05) in pairs (chi-square test).

## Supplementary Materials

The following are available online at https://www.mdpi.com/2072-6643/11/9/2183/s1, Table S1: Questionnaire food items (62 items) description and foods aggregation into 25 food items, Table S2: Factor loading matrix for the two major dietary patterns identified by principal component analysis with 60 food items as input variables (excluding all kinds of fruits and all kinds of vegetables), Table S3: Factor loading matrix for the two major dietary patterns identified by principal component analysis with 25 food items as input variables, Table S4: Food Frequency Questionnaire (62-item FFQ-6) including a manual for the adjustment of consumption frequency of single items of vegetables and fruits.
