# Supplementary material for: Reproducibility of A Non-Quantitative Food Frequency Questionnaire (62-Item FFQ-6) and PCA-Driven Dietary Pattern Identification in 13–21-Year-Old Females
Source: Nutrients. 2019 Sep 11;11(9):2183. doi: 10.3390/nu11092183 (PMC6770086; doi:10.3390/nu11092183)
Supplement: Supplementary file 1 [file nutrients-11-02183-s001.pdf]

Table S1. Questionnaire food items (62 items) description and foods aggregation into 25 food items.

No	Questionnaire item (62 items) [Q number]	Food examples Food groups description	Aggregated food groups (25 items)	
			Food groups	No
1	Sugar [Q5]	Sugar added to beverages, such as tea, coffee, etc.	Sugar, sweets and snacks	1-7
2	Honey [Q6]	Honey added to dishes and added to beverages		
3	Chocolates [Q7]	Chocolate, chocolate sweets and chocolate bars		
4	Sugar confectionery [Q8]	Boiled sweets, hard caramels, jellied sweets, fudge, etc.		
5	Baked confectionery [Q9]	Biscuits, cream cakes, fruit cakes, sponge cakes, cheesecakes, doughnuts, poppy-seed cakes, muffins, croissants, etc.		
6	Ice-creams and custard [Q10]	Ice-creams and custard		
7	Savoury snacks [Q11]	Crisps, crackers, pretzels, etc.		
8	Milk and milk beverages – natural [Q12]	Milk and natural milk beverages (yoghurt, kefir, buttermilk), porridge, etc.	Milk, fermented milk drinks and curd cheese	8+9
9	Cheese curds [Q14]	Cheese curd, natural cottage cheese, soft cheese, mozzarella, cottage cheese with herbs, etc.		
10	Milk beverages – sweetened [Q13]	Fruit yoghurts, yoghurts with chocolate flakes, flavoured buttermilk, hot chocolate, etc.	Sweetened milk products	10+11
11	Flavoured cheese curds [Q15]	Flavoured curds (with fruit, chocolate, vanilla), etc.		
12	Cheese [Q16]	Hard cheese, blue cheese, processed cheese, cheese spreads, etc.	Cheese	12
13	Eggs and egg dishes [Q17]	Scrambled eggs, omelette, egg salad, cooked eggs	Eggs and egg dishes	13
14	Breakfast cereals [Q22]	Muesli, cornflakes, other cereals - sweetened or unsweetened, etc.	Breakfast cereals	14
15	Wholemeal cereals [Q18]	Wholemeal wheat or rye bread, seeded loafs, pumpernickel, wholemeal cracker bread, etc.	Whole grain products	15+16
16	Coarse groats [Q20]	Buckwheat groats, barley, brown rice, wholemeal pasta, etc.		
17	Refined cereals [Q19]	White bread, rye, wheat-rye bread, toast bread, white bread rolls, brioche, bagels, etc.	Refined grain products	17+18
18	Fine groats [Q21]	Semolina, milled barley, pasta, white rice, rice flakes, etc.		
19	Butter [Q24]	Butter	Butter and cream	19+20
20	Cream [Q26]	Single, double, sour, used as an ingredient or added to beverages		
21	Other animal fats [Q27]	Lard, pork fat, etc.	Other animal fats	21
22	Vegetable based oil [Q23]		Vegetable oils	22
23	Margarine [Q25]	Margarine for baking, frying, spreading	Other edible fats	23+24
24	Mayonnaise [Q28]	Mayonnaise and salad dressings		
25	All kinds of fruits [Q29]	All kinds of fruits	Fruits	25
25a	Stone fruit [Q30]	Apricots, cherries, nectarines, peaches, plums, grapes, etc.	NA	
25b	Kiwi and citrus fruit [Q31]	Kiwi, oranges, mandarins, grapefruit, lemons, pomelos, etc.	NA	
25c	Tropical fruits [Q32]	Pineapples, watermelon, melons, fresh dates and figs, etc.	NA	
25d	Berries [Q33]	Strawberries, raspberries, blackberries, blueberries, redcurrants, blackcurrants, etc.	NA	
25e	Bananas [Q34]	Bananas	NA	
25f	Apples and pears [Q35]	Apples, pears	NA	
25g	Avocado [Q36]		NA	
25h	Olives [Q37]		NA	
26	Dried fruit [Q38]	Raisins, dried apricots, figs, apples, prunes, etc.	Dried fruit, fruit preserves and fruit condiments	26+27
27	Fruit preserves and fruit condiments [Q39]	Fruit compote, jams, marmalades, dates, dried bananas, etc.		
28	All kinds of vegetables (potatoes not included) [Q40]	All kinds of vegetables (potatoes not included)	Vegetables	28

28a	Cruciferous vegetables [Q41]	Cabbages, Brussel sprouts, cauliflower, broccoli, kale, etc.	NA	
28b	Yellow-orange vegetables [Q42]	Carrots, peppers, etc.	NA	
28c	Leafy green vegetables [Q43]	Spinach, chicory, lettuce, rocket, leek, celery, parsley, etc.	NA	
28d	Tomatoes [Q44]	Tomatoes	NA	
28e	Gourds and squashes [Q45]	Fresh cucumber, marrow, courgettes, pumpkins, aubergines, etc.	NA	
28f	Root vegetables and others [Q46]	Parsnip, beetroots, onion, garlic, celeriac, radishes, turnip, salads and mixed vegetables, etc.	NA	
28g	Fresh and tinned legumes [Q47]	Corn, green peas, green beans, etc.	NA	
29	Dry and processed pulses [Q48]	Beans (fava, butter kidney, broad, French, green), soya, peas, chickpea and processed pulses (baked beans, hummus, other bread spreads)	Dry and processed pulses	29
30	Potatoes [Q49]	Boiled, baked, French fries, potato rosti, gnocchi, etc.	Potatoes	30
31<br>32	Nuts and nut spreads [Q50]<br>Seeds and bran [Q51]	Peanuts, hazelnuts, walnuts, cashews, coconuts, chestnuts, peanut butter, chocolate-nut spread, etc.<br>Pumpkin seeds, sesame seeds, sunflower seeds, wheat germs, wheat bran, etc.	Nuts and seeds	31+32
33<br>34<br>35	Sausages, bacon, reconstituted meat [Q52]<br>High quality cured meats [Q53]<br>Offal products [Q54]	Sausages, meat loaf, hot-dogs, smoked sausages, bacon, etc.<br>Ham, poultry and pork-beef good quality cold meats, etc.<br>Liver, blood sausage, sweetbread, liver pate, etc.	Processed meats	33-35
36<br>37	Red meat [Q55]<br>Venison [Q57]	Pork, beef, veal, etc.<br>Wild boar, venison, quail, mallard, hare, etc.	Red meat and venison	36+37
38	Poultry and rabbit [Q56]		White meat	38
39<br>40	Lean fish [Q58]<br>Oily fish [Q59]	Pollock, cod, perch, hake, carp to 1 kg, tuna, panga, trout, etc.<br>Salmon, sardines, herring, mackerel, eel, large carp, etc.	Fish	39+40
41<br>42	Fruit juices and nectars [Q60]<br>Vegetable and vegetable-fruit juices [Q61]	Mixed fruit juice, orange, grapefruit, apple, pear, grape, blackcurrant, cherry juice, etc.<br>Mixed vegetable juice, tomato, carrot and carrot-fruit juice, etc.	Juices	41+42
43<br>44	Sweetened beverages [Q62]<br>Energy drinks [Q63]		Sweetened beverages and energy drinks	43+44
45	Beer [Q64]	Beer	Alcohol	45-47
46	Wine and cocktails [Q65]	Wine and cocktails		
47	Spirits [Q66]	Vodka and other spirits		

NA – not applied.

Table S2. Factor loading matrix for the two major dietary patterns (DPs) identified by principal component analysis with 60 food items as input variables (excluding all kinds of fruits and all kinds of vegetables).

**Food groups**[1]	**Test**		**Retest**	
	**60-item-DP1**	**60-item-DP2**	**60-item-DP1**	**60-item-DP2**
Tropical fruits	**0.75**	0.07	0.58	0.32
Bananas	**0.73**	0.13	0.72	0.13
Stone fruit	**0.72**	0.09	0.58	0.32
Olives	**0.71**	-0.07	0.79	-0.08
Avocado	**0.67**	-0.22	0.76	-0.20
Berries	**0.66**	0.11	0.66	0.08
Apples and pears	**0.65**	0.01	0.58	0.18
Kiwi and citrus fruit	**0.64**	0.11	0.45	0.41
Sweetened beverages	**0.62**	0.34	0.66	0.35
Nuts and nut spreads	**0.62**	-0.02	0.56	0.08
Vegetable and vegetable-fruit juices	**0.56**	0.01	0.11	0.52
Sugar confectionery	**0.54**	0.36	0.69	-0.08
Milk beverages – sweetened	**0.53**	0.12	0.29	0.07
Fruit juices and nectars	**0.52**	0.13	0.20	0.55
Dried fruit	**0.52**	**-0.41**	0.73	-0.21
Fruit preserves and fruit condiments	**0.51**	0.28	0.65	0.15
Savoury snacks	**0.44**	0.33	0.65	-0.07
Coarse groats	**0.42**	-0.08	-0.02	0.31
Leafy green vegetables	0.32	**0.60**	0.40	0.40
Potatoes	0.37	**0.59**	0.47	0.27
Baked confectionery	0.28	**0.57**	0.47	0.11
Gourds and squashes	0.35	**0.55**	0.50	0.27
Sausages, bacon, reconstituted meat	0.19	**0.55**	0.26	0.46
Tomatoes	0.18	**0.52**	0.26	0.34
Refined cereals	-0.02	**0.49**	0.12	0.27
Sugar	-0.30	**0.49**	0.06	0.01
Butter	0.09	**0.49**	0.15	0.41
Ice-creams and custard	0.24	**0.45**	0.48	-0.13
Cruciferous vegetables	0.35	**0.42**	0.22	0.48
Honey	-0.04	**0.40**	0.11	0.29
Fresh and tinned legumes	0.31	-0.32	0.65	0.04
Yellow-orange vegetables	0.39	0.38	0.62	0.33
Chocolates	0.39	0.33	0.60	0.03
Oily fish	0.34	-0.24	0.58	-0.05
Dry and processed pulses	0.20	-0.02	0.43	0.14
Milk and milk beverages – natural	0.23	-0.23	0.41	-0.14
Wine and cocktails	0.08	0.17	0.00	0.55
Margarine	0.00	0.21	-0.07	0.54
Beer	0.21	0.30	0.05	0.52
Cream	0.12	0.20	-0.13	0.45
Other animal fats	-0.08	0.16	0.01	0.45
High quality cured meats	0.17	0.13	0.07	0.43
Eggs and egg dishes	-0.05	0.25	-0.06	0.40
Venison	0.05	0.03	0.08	0.38
Root vegetables and others	0.17	0.32	0.34	0.37
Spirits	-0.06	0.13	-0.06	0.36
Vegetable based oil	0.04	0.25	-0.20	0.35
Mayonnaise	0.00	0.38	0.01	0.34
Cheese	-0.02	0.33	-0.08	0.32
Wholemeal cereals	0.14	0.26	0.27	0.32
Fine groats	0.06	0.12	-0.02	0.29
Seeds and bran	0.10	0.03	0.17	0.28

Red meat	-0.07	0.32	0.10	0.27
Brakfast cereals	0.37	-0.23	0.22	0.23
Cheese curds	0.21	-0.02	-0.02	0.21
Lean fish	0.16	-0.22	0.17	0.18
Offal products	0.03	0.16	0.01	0.11
Flavoured cheese curds	0.27	0.16	0.29	0.06
Poultry and rabbit	-0.01	0.04	-0.07	0.04
Variance explained (%)	15.9	7.5	18.0	7.8
Total variance explained (%)	23.4		25.8	

[1]Sorted by loadings of factor 1 (test), then factor 2 (test), factor 1 (retest) and factor 2 (retest).

Table S3. Factor loading matrix for the two major dietary patterns identified by principal component analysis with 25 food items as input variables.

Food groups[1]	Test		Retest	
	25-item-DP1	25-item-DP2	25-item-DP1	25-item-DP2
Refined grain products	**0.68**	-0.19	0.24	0.30
Processed meats	**0.63**	0.00	0.37	0.33
Sweetened beverages and energy drinks	**0.61**	0.21	0.79	0.11
Sugar, sweets and snacks	**0.59**	0.00	0.55	0.09
Potatoes	**0.59**	0.21	0.41	0.25
Butter and cream	**0.57**	0.07	0.13	0.65
Other edible fats	**0.48**	0.07	-0.02	0.67
Vegetable oils	**0.46**	0.09	-0.26	0.59
Alcohol	**0.40**	0.13	0.32	0.17
Fruits	0.00	**0.67**	0.16	0.40
Breakfast cereals	-0.03	**0.66**	0.27	0.35
Juices	0.36	**0.62**	0.25	0.66
Dried fruit, fruit preserves and fruit condiments	0.05	**0.53**	0.62	0.13
Vegetables	0.14	**0.53**	0.04	0.45
Nuts and seeds	0.13	**0.51**	0.69	0.12
Milk, fermented milk drinks and curd cheese	-0.13	**0.50**	0.06	0.27
Sweetened milk products	0.30	**0.45**	0.32	0.17
Dry and processed pulses	0.13	0.20	0.63	-0.03
Fish	-0.11	0.28	0.52	-0.13
Chesse	0.14	0.27	-0.23	0.64
Eggs and egg dishes	0.35	-0.03	-0.01	0.44
Whole grain products	0.04	0.39	0.21	0.40
Other animal fats	0.11	-0.13	0.18	0.35
White meat	0.26	0.08	0.03	0.08
Red meat and venison	0.38	-0.28	0.35	-0.02
Variance explained (%)	16.1	10.5	17.7	10.5
Total variance explained (%)	26.6		28.2	

[1]sorted by loadings of factor 1 (test), then factor 2 (test), factor 1 (retest) and factor 2 (retest).

Table S4.
Food Frequency Questionnaire (62-item FFQ-6)

We are conducting research on nutrition and health. Please answer all the questions as best as you can. If you cannot answer, please specify approximately. This answer will be more helpful to us than the lack of response. The information provided is confidential and will be used only for scientific purposes.

Comment and instructions for the interviewer. The interviewer reads the question, possible answers and notes the answer chosen by the respondent.

1. Respondent code (to be completed by the interviewer) ☐☐☐☐

2. Gender 1. Male ☐ 2. Female ☐

3. Date of the interview (day/month/year) ☐☐ ☐☐ ☐☐☐☐

4. Date of birth (day/month/year) ☐☐ ☐☐ ☐☐☐☐

Please tell me how often did you eat the following products?
Please choose one answer that best describes your diet in the past 12 months.
Please consider all meals and snacking, eaten at home and when you are out.

**In the past 12 months, how often did you eat or drink:**	Never or almost never	Once a month or less	Several times a month	Several times a week	Daily	Several times a day
Codes of consumption frequency:	*(1)*	*(2)*	*(3)*	*(4)*	*(5)*	*(6)*
**Sweets and snacks**						
**5. Sugar** for sweetening beverages						
**6. Honey** for sweetening dishes and beverages						
**7. Chocolate, chocolate sweets and chocolate bars**						
**8. Sugar confectionery**, e.g. boiled sweets, hard caramels, jellied sweets, fudge						
**9. Baked confectionery**, e.g. biscuits, cream cakes, fruit cakes, sponge cakes, cheesecakes, doughnuts, poppy-seed cakes, muffins, croissants						
**10. Ice-creams and custard**						
**11. Savoury snacks,** e.g. crisps, crackers, pretzels						

Authors: Lidia Wadolowska and Ewa Niedzwiedzka

**In the past 12 months, how often did you eat or drink:**	Never or almost never	Once a month or less	Several times a month	Several times a week	Daily	Several times a day
Codes of consumption frequency:	*(1)*	*(2)*	*(3)*	*(4)*	*(5)*	*(6)*
**Dairy and eggs**						
**12. Milk and milk beverages – natural**, e.g. milk and natural milk beverages (yoghurt, kefir, buttermilk), porridge						
**13. Milk beverages – sweetened**, e.g. fruit yoghurts, yoghurts with chocolate flakes, flavoured buttermilk, hot chocolate						
**14. Cheese curds**, e.g. cheese curd, natural cottage cheese, soft cheese, mozzarella, cottage cheese with herbs						
**15. Flavoured cheese curds**, e.g. with fruit, chocolate, vanilla						
**16. Cheese**, e.g. hard cheese, blue cheese, processed cheese, cheese spreads						
**17. Eggs and egg dishes,** e.g. scrambled eggs, omelette, egg salad, cooked eggs						
**Cereals**						
**18. Wholemeal cereals**, e.g. wholemeal wheat or rye bread, seeded loafs, pumpernickel, wholemeal cracker bread						
**19. Refined cereals,** e.g. white bread, rye, wheat-rye bread, toast bread, white bread rolls, brioche, bagels						
**20. Coarse groats**, e.g. buckwheat groats, barley, **brown rice, wholemeal pasta**						
**21. Fine groats**, e.g. semolina, milled barley, **pasta, white rice**, rice flakes						
**22. Breakfast cereals, e.g.** muesli, cornflakes, other cereals - sweetened or unsweetened						

**In the past 12 months, how often did you eat or drink:**	Never or almost never	Once a month or less	Several times a month	Several times a week	Daily	Several times a day
Codes of consumption frequency:	*(1)*	*(2)*	*(3)*	*(4)*	*(5)*	*(6)*
**Fats**						
**23. Vegetable based oil,** all kinds						
**24. Butter,** all kinds						
**25. Margarine** for baking, frying, spreading, all kinds						
**26. Cream,** e.g. single, double, sour, used as an ingredient or added to beverages						
**27. Other animal fats**, e.g. lard, pork fat						
**28. Mayonnaise and salad dressings**, all kinds						
**Fruit**						
**29. Fruit, all kinds** Ask separately for total fruit and then for different types of fruit						
**30. Stone fruit**, e.g. apricots, cherries, nectarines, peaches, plums, grapes						
**31. Kiwi and citrus fruit**, e.g. kiwi, oranges, mandarins, grapefruit, lemons, pomelos						
**32. Tropical fruits**, e.g. pineapples, watermelon, melons, fresh dates and figs						
**33. Berries**, e.g. strawberries, raspberries, blackberries, blueberries, redcurrants, blackcurrants						
**34. Bananas**						
**35. Apples and pears**						
**36. Avocado**						
**37. Olives**						
**38. Dried fruit**, e.g. raisins, dried apricots, figs, apples, prunes						
**39. Fruit preserves and fruit condiments**, e.g. fruit compote, jams, marmalades, dates, dried bananas						

**In the past 12 months, how often did you eat or drink:**	Never or almost never	Once a month or less	Several times a month	Several times a week	Daily	Several times a day
Codes of consumption frequency:	*(1)*	*(2)*	*(3)*	*(4)*	*(5)*	*(6)*
**Vegetables and legumes**						
**40. Vegetables, all kinds (potatoes not included)** Ask separately for total vegetables and then for different types of vegetables						
**41. Cruciferous vegetables**, e.g. cabbages, Brussel sprouts, cauliflower, broccoli, kale						
**42. Yellow-orange vegetables**, e.g. carrots, peppers						
**43. Leafy green vegetables**, e.g. spinach, chicory, lettuce, rocket, leek, celery, parsley						
**44. Tomatoes**						
**45. Gourds and squashes**, e.g. fresh cucumber, marrow, courgettes, pumpkins, aubergines						
**46. Root vegetables and others**, e.g. parsnip, beetroots, onion, garlic, celeriac, radishes, turnip, salads and mixed vegetables						
**47. Fresh and tinned legumes**, e.g. corn, green peas, green beans						
**48. Dry and processed pulses**, e.g. beans (fava, butter kidney, broad, French, green), soya, peas, chickpea and processed pulses (baked beans, hummus, other bread spreads)						
**49. Potatoes,** in various forms, e.g. boiled, baked, French fries, potato rosti, gnocchi						
**50. Nuts and nut spreads**, e.g. peanuts, hazelnuts, walnuts, cashews, coconuts, chestnuts, peanut butter, chocolate-nut spread						
**51. Seeds and bran**, e.g. pumpkin seeds, sesame seeds, sunflower seeds, wheat germs, wheat bran						

**In the past 12 months, how often did you eat or drink:**	Never or almost never	Once a month or less	Several times a month	Several times a week	Daily	Several times a day
Codes of consumption frequency:	*(1)*	*(2)*	*(3)*	*(4)*	*(5)*	*(6)*
**Meat and fish**						
52. **Sausages, bacon, reconstituted meat**, e.g. sausages, meat loaf, hot-dogs, smoked sausages, bacon						
53. **High quality cured meats**, e.g. ham, poultry and pork-beef good quality cold meats						
54. **Offal products**, e.g. liver, blood sausage, sweetbread, liver pate						
55. **Red meat**, e.g. pork, beef, veal						
56. **Poultry and rabbit**, e.g. chicken, duck, turkey						
57. **Venison**, e.g. wild boar, venison, quail, mallard, hare						
58. **Lean fish**, e.g. pollock, cod, perch, hake, carp to 1 kg, tuna, panga, trout						
59. **Oily fish**, e.g. salmon, sardines, herring, mackerel, eel, large carp						
**Beverages**						
60. **Fruit juices and nectars,** e.g. mixed fruit juice, orange, grapefruit, apple, pear, grape, blackcurrant, cherry juice						
61. **Vegetable and vegetable-fruit juices,** e.g. mixed vegetable juice, tomato, carrot and carrot-fruit juice						
62. **Energy drinks**<br>The list of products should be prepared by the researcher						
63. **Sweetened beverages,** such as Fanta, Coca-cola, Mirinda, Sprite etc.						
64. **Beer**						
65. **Wine and cocktails**						
66. **Vodka and other spirits**						

Thank you for answering and your time

A manual for the adjustment of consumption frequency of single items of vegetables and fruits

First step:

The consumption frequency collected with the FFQ-6 questionnaire (six categories to choose from) should be converted into the daily frequency of consumption [times/day] as follows:

Categories	Daily frequency of consumption [times/day]
(1) never or very rarely	0
(2) once a month or less	0.025
(3) several times a month	0.1
(4) several times a week	0.571
(5) daily	1
(6) a few times a day	2

Second step:

Based on the data on consumption frequency of vegetables and fruits in general (questions Q40 and Q29, respectively), **for each respondent individual correction coefficients** (weights) should be determined to adjust the consumption frequency of single items of vegetables and fruits collected with separate questions (Q41-47 and Q30-37, respectively), according to the formulas:

correction coefficient for vegetables (weight) = consumption frequency of **vegetables in general** (all kinds) divided by **the sum of the consumption frequency of seven single items** of vegetables

(Q40) [times/day]/ (Q41+Q42+Q43+Q44+Q45+Q46+Q47) [times/day]

where: Vegetables, all kinds [Q40], Cruciferous vegetables [Q41], Yellow-orange vegetables [Q42], Leafy green vegetables [Q43], Tomatoes [Q44], Gourds and squashes [Q45], Root vegetables and others [Q46], Fresh and tinned legumes [Q47]

correction coefficient for fruits (weight) = consumption frequency of **fruits in general** (all kinds) divided by **the sum of the consumption frequency of eight single items** of fruits

(Q29) [times/day] / (Q30+Q31+Q32+Q33+Q34+Q35+Q36+Q37) [times/day]

where: Fruits, all kinds [Q29], Stone fruit [Q30], Kiwi and citrus fruit [Q31], Tropical fruits [Q32], Berries [Q33], Bananas [Q34], Apples and pears [Q35], Avocado [Q36], Olives [Q37]

Third step:

Adjusted frequency of consumption for each of seven single items of vegetables (or eight single items of fruits) should be calculated by **multiplying the crude consumption frequency of a single item** of vegetables (or fruits) [times/day] **and the variable with individual correction coefficients** (weights) for vegetables (or fruits) estimated for each respondent.

Note that, for example, the sum of the adjusted frequencies of consumption of seven single items of vegetables [times/day] should equal the consumption frequency of vegetables in general (all kinds) [times/day] for each respondent; the same for fruits.

The example of the above calculations in STATISTICA software (StatSoft, Krakow, Poland) is available on request sent to the corresponding author: joanna.kowalkowska@uwm.edu.pl

Authors of the manual: Lidia Wadolowska, Ewa Niedzwiedzka and Joanna Kowalkowska
